# Predicting changes in osmolality

**DOI:** 10.7554/eLife.74551

**Published:** 2021-11-18

**Authors:** Zhe Yang, Tongtong Wang, Yuki Oka

**Affiliations:** 1 Division of Biology and Biological Engineering, California Institute of Technology Pasadena United States

**Keywords:** vasopressin, presystemic, feedforward, osmolality, water balance, fiber photometry, Mouse

## Abstract

Two neural circuits control the release of vasopressin in response to eating and drinking before there are any detectable changes in blood water levels.

**Related research article** Kim A, Madara JC, Wu C, Andermann ML, Lowell BB. 2021. Neural basis for regulation of vasopressin secretion by anticipated disturbances in osmolality. *eLife* **10**:e66609. doi: 10.7554/eLife.66609

The balance between water and solutes in our blood, known as osmolality, must be tightly controlled for our bodies to work properly. Both eating and drinking have profound effects on osmolality in our body. For example, after several bites of food the brain rapidly triggers a feeling of thirst to increase our uptake of water (Leib et al., 2017; Matsuda et al., 2017). In addition, when fluid balance is disturbed, the brain releases a hormone called vasopressin that travels to the kidneys to reduce the excretion of water (Geelen et al., 1984; Thrasher et al., 1981). While much is known about how the brain controls drinking behavior, it is less clear how it regulates the hormonal response.

Vasopressin is primarily secreted by Arginine-vasopressin (AVP) neurons in the supraoptic and paraventricular nucleus of the hypothalamus. These neurons not only respond to actual disturbances in water balance, but also anticipate future osmotic changes that occur after eating and drinking. In 2017, a group of researchers discovered that AVP neurons respond to food and water by rapidly decreasing or increasing their activity, respectively, before there are any detectable changes in osmolality (Mandelblat-Cerf et al., 2017). Now, in eLife, researchers from Harvard Medical School – including Angela Kim as first author and corresponding author Bradford Lowell – report the neural pathways underlying this drinking- and feeding-induced regulation of vasopressin (Kim et al., 2021).

AVP neurons receive signals from the lamina terminalis, a brain structure that detects changes in osmolality and modulates thirst and water retention (McKinley, 2003). Using virus tracing techniques, the team (which includes some of the researchers involved in the 2017 study) mapped neurons in the lamina terminalis that are directly connected to AVP neurons in mice. This revealed that excitatory and inhibitory neurons in two regions of the lamina terminalis (called MnPO and OVLT) send direct inputs to AVP neurons.

Kim et al. then examined whether these neurons in the lamina terminalis responded to drinking and water-predicting cues (such as seeing a bowl of water being placed down; Figure 1). Excitatory neurons that drive thirst and stimulate vasopressin release were rapidly suppressed by both drinking and water-predictive cues before there were any detectable changes in blood osmolality. Conversely, inhibitory neurons showed the opposite response, and were activated following bowl placement and water consumption. This suggests that excitatory and inhibitory neurons in the lamina terminalis help anticipate future osmotic changes by reducing the activity of AVP neurons in response to drinking and water-predictive cues.

**Figure 1.**
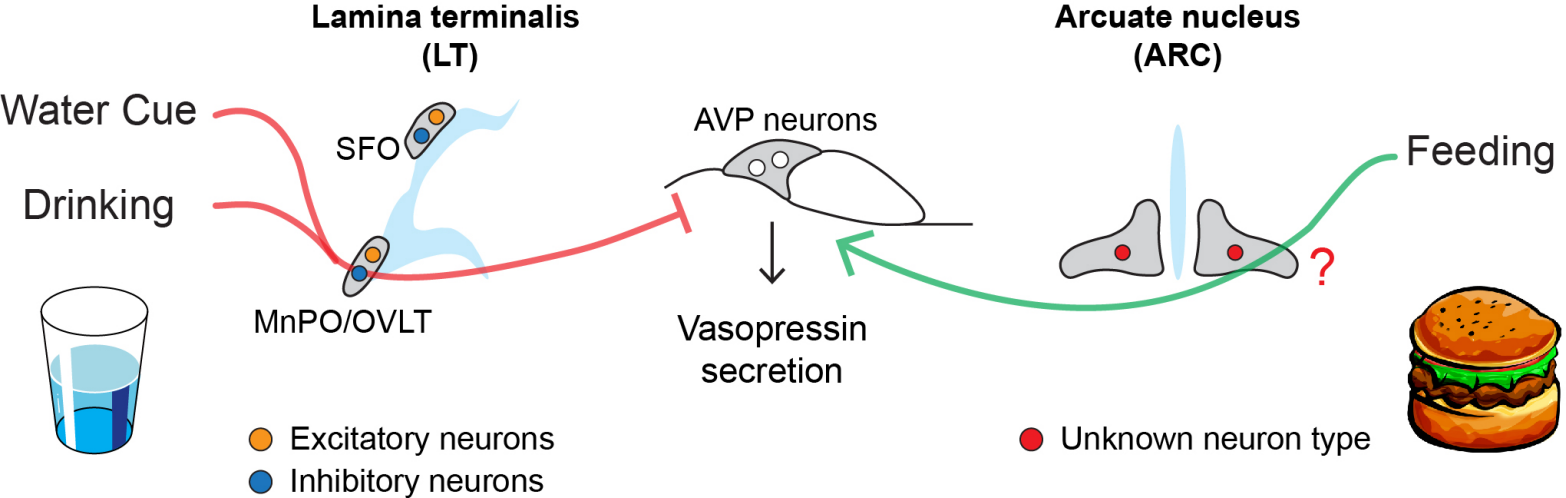
How drinking and eating alter the activity of AVP neurons. AVP neurons (middle) help maintain osmolality by releasing a hormone called vasopressin, which reduces the amount of fluids excreted from the kidneys. Eating and drinking have been shown to alter the activity of AVP neurons before there are any detectable changes in blood osmolality. Water cues (such as the presence of a glass) and drinking suppress the release of vasopressin (red line) by activating inhibitory neurons (blue circle) in the MnPO and OVLT regions of the lamina terminalis. Eating, on the other hand, stimulates AVP neurons to release vasopressin (green line) through an unknown population of neurons (red circle) in the arcuate nucleus, the region of the brain that regulates hunger. These neural circuits allow the body to react quickly to the osmotic changes caused by eating and drinking before the balance of fluids in our blood is disrupted.

Further experiments showed that food intake – but not food-predicting cues – stimulates AVP neurons to release vasopressin prior to an increase in blood osmolality. However, Kim et al. found that neurons in the lamina terminalis are unlikely to be involved in this process, as they did not respond to food consumption as quickly as AVP neurons. Instead, they discovered that these feeding-induced signals came from an undefined neuronal population in the arcuate nucleus, the hunger center in the brain that houses the neurons that promote and inhibit feeding (Figure 1; Atasoy et al., 2012). Unlike other neurons involved in hunger, these cells did not appear to respond to food-predicting cues. Molecular data on the different cell types in the arcuate nucleus could be used to identify this new population, potentially revealing a new hunger-related neural mechanism (Campbell et al., 2017).

Taken together, the findings of Kim et al. reveal that eating and drinking alter the activity of AVP neurons via two distinct neural circuits (Figure 1). There are, however, a few limitations to this study. For instance, the regulation of lamina terminalis neurons and vasopressin is inseparable. Indeed, manipulation of the lamina terminalis neurons inevitably changes thirst drive, water intake and the activity of AVP neurons. This makes it difficult to pinpoint the source of predictive signals in AVP neurons.

Another question has to do with the physiological significance of the anticipatory regulation of lamina terminalis neurons and AVP neurons. If water-predicting cues suppress excitatory neurons in the lamina terminalis, how does the brain maintain the desire to drink? This issue is particularly important for the thirst system since thirst-driving neurons can have acute effects on drinking behavior (Augustine et al., 2020). It is possible that the lamina terminalis regulates thirst and vasopressin secretion through different populations of neurons. Future work could investigate if the neurons directly connected to AVP neurons are different to the ones that drive thirst. Identifying the individual components of the behavioral and hormonal response may provide new insights into how the brain regulates the uptake and excretion of fluids.
